# Mental disorders and mental health symptoms during imprisonment: A three-year follow-up study

**DOI:** 10.1371/journal.pone.0213711

**Published:** 2019-03-14

**Authors:** Caroline Gabrysch, Rosemarie Fritsch, Stefan Priebe, Adrian P. Mundt

**Affiliations:** 1 Department of Psychiatry, Charité Universitätsmedizin Berlin, Berlin, Germany; 2 Department of Psychiatry and Mental Health, Hospital Clínico Universidad de Chile, Santiago, Chile; 3 Department of Social and Community Psychiatry, Queen Mary University of London, London, United Kingdom; 4 Medical Faculty, Universidad Diego Portales, Santiago, Chile; 5 Medical School, Universidad San Sebastián, Puerto Montt, Chile; University of South Florida, UNITED STATES

## Abstract

**Background:**

Data on the course of mental disorders during imprisonment are scarce. Longitudinal studies from high-income Western countries point to improvements of symptoms over time. The aim of the present study was to assess mental disorders and symptoms three years after baseline evaluation at imprisonment and to determine predictors of change in a South American prison context.

**Methods:**

Consecutively admitted prisoners in Santiago de Chile were assessed at intake and reassessed after three years using the Mini International Neuropsychiatric Interview and the Symptom-Check-List 90 Revised (SCL-90-R). The global severity index (GSI) was calculated with standard deviations (SD) and compared using paired t-tests. The prevalence of mental disorders at baseline and at follow-up were compared using McNemar tests. Analyses of variance were conducted to evaluate whether prespecified socio-demographic variables and disorders at baseline predicted symptom change at follow-up.

**Results:**

73 (94%) out of 78 prisoners participated. The prevalence of major mental illnesses was lower at follow-up: *47* (64%) at intake vs. *23* (32%) at follow-up had major depression (*p*<0.001); *22* (30%) at intake vs. *10* (14%) at follow-up had psychosis (*p* = 0.008). The mean GSI improved from 1.97 (SD 0.65) at intake to 1.16 (SD 0.82) at follow-up (*p*<0.001). Depression at baseline (*F* = 9.39; $\eta_{p}^{2}$ = 0.137; *β* = -0.67; *p* = 0.003) and working or studying during imprisonment (*F* = 10.61; $\eta_{p}^{2}$ = 0.152; *β* = -0.71; *p* = 0.002) were associated with strong improvement of the GSI at follow-up, whereas psychosis at intake was associated with relatively small symptom improvement (*F* = 12.11; $\eta_{p}^{2}$ = 0.17; *β* = 0.81; *p* = 0.001).

**Conclusions:**

In a resource poor prison context in South America, mental health symptoms and disorders improve considerably over three years during imprisonment. This applies especially to people with depression at intake. Offers to work or study during imprisonment may improve mental health outcomes.

## Introduction

The number of imprisoned people has increased worldwide over the past two decades and this is especially pronounced in South American countries [1]. The increase of prison populations is associated with a decreasing number of psychiatric hospital beds in South America [2]. In 2015, there were 247 people imprisoned per 100,000 population in Chile compared to 144 worldwide [1]. Occupancy rates in prisons are above 110% and conditions are poor [1, 3]. The prevalence of severe mental illness among prison populations worldwide is high with one in seven prisoners estimated to have major depression or psychosis [4]. Rates for affective disorders and suicide risk were particularly high in newly admitted prison populations in Chile compared to high-income countries [5], and mental disorders and symptoms remain frequently unrecognized and untreated.

Fifteen longitudinal studies on changes in mental health symptoms within the prison environment were included in a recent review [6]. All of these studies were conducted in high-income Western countries and reported either symptoms or diagnoses with predominantly short follow-up periods [6]. The studies that used standardized diagnostic interviews [7, 8] applied self-rating instruments at screening which may have had negative effects on the representativeness of the samples. Despite the evidence being reported to be inconclusive overall, the reviewed studies suggested an improvement in mental health symptoms of sentenced prisoners over time [6]. Mental health symptoms seem to be highest at intake to prison [7, 9–11] and improvement over the course of imprisonment has been shown for consecutively imprisoned people with major depression at intake in Chile [12].

The aim of the present study was to assess the course of mental disorders and symptoms in a South American prison context over a three-year follow-up period and to identify factors that predict improvements.

## Materials and methods

### Sample

We conducted a longitudinal observational follow-up study on consecutively admitted individuals to three prisons in Chile. Prisoners were assessed at baseline and three years later.

The inclusion criteria for follow-up assessments were: having participated in the baseline study in 2013 and remaining imprisoned or being consecutively imprisoned at follow-up (which was conducted between November 2016 and January 2017); living in the metropolitan region of Santiago de Chile; and having capacity to provide informed consent.

At baseline in 2013, 427 randomly selected prisoners were assessed within their first weeks of imprisonment in three central remand prison facilities of the metropolitan region of Santiago de Chile [5]. After three years, 78 were still imprisoned or had been re-imprisoned. All 78 imprisoned individuals who met the inclusion criteria at follow-up were approached for an interview. Participants were located in 8 different prison facilities for remand and sentenced prisoners: Centro de Detención Preventiva (CDP) Santiago Sur, CDP Santiago Uno, Centro Cumplimiento Penitenciario (CCP) Colina Uno, CCP Colina Dos, CDP Puente Alto, Centro Penitenciario Femenino (CPF) San Joaquin, CPF San Miguel and the nocturnal reclusion facility Centro de Educación y Trabajo.

### Instruments

Age, marital status, employment status, educational level, social network, legal status, previous imprisonments, psychological and psychiatric treatment were assessed using structured questions. Marital status was categorized in the exclusive categories: single, married, co-residing, separated, divorced and widowed. Educational level was categorized according to the International Standard Classification of Education (ISCED), comprising levels 4, 5 and 6 (postsecondary education, university and doctorate degrees) to one level [13]. Legal status was dichotomized to remand and sentenced, and the type of criminal offence was recorded. The pattern of imprisonment(s) during the three-year follow-up period was obtained from prison administration as continuous or intermittent.

The Spanish version of the Mini-International Neuropsychiatric Interview (MINI) was used to establish diagnoses in accordance with the Diagnostic and Statistical Manual of Mental Disorders (DSM-IV). The MINI is a fully structured interview developed by Sheehan et al. (1998). Since it was first used to assess the mental health status of a prison population in 2004, it has been applied in several countries [14–18]. The module for borderline personality disorder of the Structured Clinical Interview for DSM-IV (SCID-II) was also included in the interview [19].

The Spanish revised version of the Symptom Check list with 90 items (SCL-90-R) was used to assess the severity of psychological symptoms. Study participants select from a 5-point Likert-type scale that ranges from ‘*not at all’* to ‘*extremely’*. The instrument covers symptom dimensions including Somatization, Obsessions–Compulsions, Interpersonal Sensitivity, Depression, Anxiety, Hostility, Phobic Anxiety, Paranoid Ideation and Psychoticism. The SCL-90-R was found to be a reliable and valid instrument used for the screening of imprisoned people for mental disorders and has previously been used in prison populations [10, 20, 21].

### Procedure

The procedure for the baseline assessments has been described in detail elsewhere [5]. Potential participants fulfilling the inclusion criteria were approached by the prison staff and led to private rooms for the interviews. To ensure confidentiality, interviews were held by members of the research team, in absence of prison staff and in private rooms. The field team consisted of two psychologists, one psychology student and one doctoral student in medicine. They were trained by two senior consultant psychiatrists in applying the structured and diagnostic interviews with volunteers until satisfactory consensus between the interviewers was reached. To further ensure consensus, the first 30 interviews with imprisoned individuals were conducted by two members of the field team, of which one was the active interviewer. The role of the active interviewer was alternated. Ratings and diagnoses were then compared and discussed.

Written and oral informed consent was obtained from every participant prior to inclusion in the follow-up assessment. The study information contained a statement that study participation was voluntary and independent from all legal issues or other benefits. After resolving all questions, it was emphasized that the consent to participate could be withdrawn at any later point of time without further explanations and without any consequences. Capacity to provide informed consent was deducted from the capacity to sufficiently concentrate on the oral and written study information and to reproduce parts of it in order to show that contents and procedures were understood. No treatments were offered to participants of the study.

The study was approved by the institutional ethics review board of the University Hospital of the University of Chile (Acta de aprobación Número 10 del 06 de abril 2016, Comité Ético Científico, Hospital Clínico Universidad de Chile), by the Ministry of Justice (Oficio Número 2478, del 19 de abril 2016, Jefa División Reinserción Social) and by the national prison administration, Gendarmería de Chile (Oficio Numero 671/2016 del 9 Nov 2016, Director Regional Metropolitano Gendarmería de Chile).

### Analyses

Socio-demographic characteristics and prevalence rates of mental disorders were calculated as per cent values at baseline and at follow-up. The age and the scores on the SCL-90-R were calculated as means with standard deviations. McNemar tests were conducted to test whether, and how, the prevalence of mental disorders differed between baseline and follow-up. A paired sample t-test was used to assess the difference between the mean GSI and subscale scores on the SCL-90-R symptom score at baseline and follow-up. A paired sample t-test was also used to test whether mean GSI scores differed for groups of diagnostic entities at baseline. Analysis of variance with parameter estimates was used to assess the association of prespecified socio-demographic factors and groups of diagnostic entities at baseline with change of the GSI. We included gender, educational level, which was dichotomized for this purpose as low (ISCED 0–1) and other (ISCED 2–6), any mental health treatment, working (including working for remuneration or studying), legal status (remand vs. sentenced) and consecutive vs. intermittent imprisonments during follow-up (interval(s) outside prison during follow-up). Psychiatric morbidities at baseline were included. *P*-values <0.05 were considered statistically significant. All statistical data analyses were conducted using SPSS version 24.

## Results

At three-year follow-up 78 individuals were eligible for participation in the follow-up study. Three did not respond on the third call, one did not give consent and one was ineligible due to acute psychotic symptoms incompatible with the interview. The non-response rate was therefore 3.8% and the refusal rate was 2.5%, adding up to 6.4%. Seventy-three prisoners were included in the follow-up study, of which *37* (51%) had been released from prison at least once since the baseline assessment. All participants in the study completed the MINI, with 72 participants completing the SCL-90-R (data missing for one participant).

### Socio-demographic characteristics

Socio-demographic characteristics of the sample are reported in Table 1 as total numbers and as percentage values. Most imprisoned individuals were non-migrant Chileans, male, single and had low educational levels. Fifty-three (73%) had at least one child. Three years after the baseline assessments 61 (84%) were sentenced, with almost half of these sentences being for violent crimes. Apart from three women in nocturnal imprisonment, all were in complete imprisonment. At follow-up, over 90% received visitors, more than half of the sample at least weekly. Fifty-two participants (71%) did not work for income, 16 (22%) were in paid employment, and two (3%) were in full time study. Ten (14%) prisoners had received specialized mental health treatment at least once within the follow-up period, one as an inpatient and 13 (18%) reported using prescribed medication on a regular basis.

**Table 1:** Socio-demographic characteristics of people followed-up after three years in the prison system of Santiago de Chile.

	Total Sample		Male		Female	
	*N = 73*	%	*n = 53*	%	*n = 20*	%
Median age (min/max)	30 (21/77)		30 (21/77)		30.5 (22/62)	
Chilean	72	99	52	98	20	100
Non-Chilean	1	1	1	2	0	-
**Marital status**						
Married	5	7	4	8	1	5
Co-residing	4	6	4	8	0	-
Divorced	3	4	3	6	0	-
Separated	2	3	0	-	2	10
Single	57	78	41	77	16	80
Widowed	2	3	1	2	1	5
**Number of children**						
None	20	27	17	32	3	15
1	24	33	18	34	6	30
2	11	15	8	15	3	15
3–6	18	25	10	19	8	40
**Legal situation**						
Pre-trial	12	16	10	19	2	10
Sentenced	61	84	43	81	18	90
**Type of imprisonment**						
Complete reclusion	70	96	53	100	17	85
Partial reclusion	3	4	0	-	3	15
**Pattern of imprisonment**						
Consecutive	36	49	26	49	10	50
Repeatedly	37	51	27	51	10	50
**Receiving visits**						
Never	7	10	3	6	4	20
Monthly or less	27	37	18	34	9	45
Weekly	39	53	32	60	7	35
**Educational level**						
ISCED 0	15	21	12	23	3	15
ISCED 1	37	51	27	51	10	50
ISCED 2	19	26	12	23	7	35
ISCED 3	2	3	2	4	0	-
ISCED 4 to 6	0	-	0	-	0	-
**Offense category (non-excl.)**						
Robbery	20	27	16	30	4	20
Violence (incl. Homicide)	33	45	28	53	5	25
Drug possession/trafficking	14	19	4	8	10	50
Sexual crime	8	11	8	15	0	-
Other	12	16	9	17	3	15
**Current Work Situation**						
Not working for income	52	71	38	72	14	70
Working for income	16	22	10	19	6	30
Employed but not working	1	1	1	2	0	-
Studying	2	3	2	4	0	-
Other	2	3	2	4	0	-
**Mental health and psychotropic treatment**						
Any treatment	10	14	7	13	3	15
Outpatient	9	12	6	11	3	15
Inpatient	1	1	1	2	0	-
Use of medication	13	18	8	15	5	25

ISCED: International Standard Classification of Education

### Mental disorders at admission and at follow-up

DSM-IV diagnoses at admission and at follow-up as well as the outcomes of the McNemar tests are reported in Table 2. Out of *47* (64%) individuals with major depression at baseline, *17* (23%) still met the diagnostic criteria at follow-up, *and* 26 (36%) had been remitted at follow-up and there were 6 new cases. The prevalence decreased from 64% to 32% (p<0.001). The prevalence of anxiety disorders was *32* (44%) at baseline and *27* (37%) at follow-up. The prevalence of current psychotic disorders decreased from *20* (27%) at baseline to *8* (11%) at follow-up (*p* = 0.008). Personality disorders (borderline and antisocial PD) were present in *52* (71%) at baseline and in *51* individuals (70%) at follow-up. Forty-seven (64%) had substance use disorder at baseline and *36* (50%) at follow-up. There were 10 new cases of illicit drug use disorders among the 26 prisoners who did not have this disorder at baseline.

**Table 2:** The prevalence of psychiatric disorders (diagnostic groups) among prisoners at baseline and at three-year follow-up.

	Baseline				Follow-up			McNemar test			
	N = 73	Prevalence in % (95% CI)		N = 73		Prevalence in % (95% CI)		Cases at baseline and follow-up (*n)*	New cases at follow-up (*n)*	Remitted cases at follow-up (*n)*	*p*-value
Depression^a^	47	64	(53.4–75.3)	23		32	(20.5–42.5)	17	6	26	<0.001
Anxiety disorders^b^	32	44	(31.5–56.2)	27		37	(26–47.9)	18	9	14	n.s.
Psychotic disorders^c^	22	30	(19.2–39.7)	10		14	(6.8–23.3)	7	3	15	0.008
PD	52	71	(60.3–82.2)	51		70	(58.9–80.8)	42	9	10	n.s.
IDUD	47	64	(53.4–75.3)	36		50	(38.4–61.6)	29	10	21	n.s.
AUD	22	30	(19.2–41.1)	13		18	(9.6–27.4)	6	7	16	n.s.

^a^Including major depression, recurrent major depression

^b^including current panic disorder, agoraphobia, social anxiety disorder, generalized anxiety disorder; OCD = obsessive-compulsive disorder; PTSD = posttraumatic stress disorder

^c^including current psychotic disorder, current psychotic mood disorder; PD = personality disorder (including borderline personality disorder and antisocial personality disorder); IDUD = illicit drug use disorder (including drug dependence and drug abuse); AUD = alcohol use disorder (including alcohol dependence and alcohol abuse); n.s. = non-significant.

### Suicide risk

Suicide risk was present in 45 (62%) participants at baseline and in 35 (48%) at follow-up. Prevalence rates of high and moderate suicide risk dropped to half (moderate suicide risk from *10* to *5*, high suicide risk from *21* to *10*) while prevalence of low suicide risk increased from *14* (19%) to *20* (27%). Eleven participants (15%) attempted suicide during the three-year imprisoned period, *9* during the current imprisonment, *7* of them had shown high suicide risk at baseline.

### Psychological symptoms

The mean values of the GSI and the nine subscale scores at baseline and follow-up are reported in Table 3. Mean symptom scores of the GSI and all subscales decreased, reaching significance on all subscales except hostility. Thirty-seven (51%) showed reliable clinical change (improvement), *35* (49%) deteriorated or did not reach the level of reliable clinical change.

**Table 3:** Symptom scores and subscales of the psychological symptoms assessed with the revised version of the Symptom Checklist 90 at baseline and at follow-up.

	Mean (±SD) at baseline	Mean (±SD) at follow-up	Paired t-test		
			t	df	*p*-value
GSI	1.6 (0.84)	1.2 (0.82)	4.32	71	<0.001
**Dimensions**					
Somatization	1.6 (0.96)	1.1 (0.91)	4.63	71	<0.001
Depression	2.0 (1.04)	1.4 (0.96)	4.53	71	<0.001
Obsessions-Compulsions	1.8 (0.87)	1.5 (0.96)	2.86	71	0.006
Interpersonal sensitivity	1.5 (0.91)	1.2 (0.89)	2.94	71	0.004
Anxiety	1.9 (1.13)	1.3 (1.01)	4.44	71	<0.001
Hostility	1.0 (1.02)	0.8 (0.84)	1.43	71	n.s.
Phobic Anxiety	1.0 (0.92)	0.6 (0.86)	3.26	71	0.002
Paranoid Ideation	1.6 (1.06)	1.3 (1.04)	2.44	71	0.017
Psychoticism	1.4 (0.89)	1.0 (0.85)	3.44	71	0.001

The mean GSI at baseline and follow-up is shown for different diagnostic groups at baseline in Table 4. All groups, except those with psychotic disorders, showed significant symptom improvements. The mean GSI scores for people with psychosis at baseline remained high at follow-up compared to groups with other diagnoses.

**Table 4:** Mean Global Severity Indices on the revised version of the symptom checklist with 90 items between baseline and at follow-up.

Diagnostic category	Mean GSI (±SD) baseline	Mean GSI (±SD) follow-up	Paired t-test		
			t	df	*p*-value
Depression^a^	2.0 (0.73)	1.4 (0.85)	4.34	45	<0.001
Anxiety disorders^b^	2.1 (0.8)	1.6 (1.0)	2.11	19	0.048
Psychotic disorders^c^	2.0 (0.8)	1.8 (0.84)	1.03	19	n.s.
PD	1.8 (0.72)	1.3 (0.8)	4.30	50	<0.001
IDUD	1.6 (0.76)	1.2 (0.8)	3.10	45	0.003
AUD	1.8 (0.88)	1.1 (0.86)	3.71	21	0.001

^a^Including major depression, recurrent major depression

^b^including current panic disorder, agoraphobia, social anxiety disorder, generalized anxiety disorder; OCD = obsessive-compulsive disorder; PTSD = posttraumatic stress disorder

^c^including current psychotic disorder, current psychotic mood disorder; PD = personality disorder (including borderline personality disorder and antisocial personality disorder); IDUD = illicit drug use disorder (including drug dependence and drug abuse); AUD = alcohol use disorder (including alcohol dependence and alcohol abuse); n.s. = non-significant.

### Factors associated with symptom change

The association between socio-demographic variables, prison related variables, psychiatric morbidities at baseline and change of the GSI are reported in Table 5. In the analysis of variance, working during imprisonment showed the strongest association with improvement of psychological symptoms; this variable explained 15.2% of the variance and -0.71 points of the mean GSI ($\eta_{p}^{2}$ = 0.152; *β* = -0.71; *p* = 0.002). Depression at baseline ($\eta_{p}^{2}$ = 0.137; *β* = -0.67; *p* = 0.003) and intermittent imprisonment ($\eta_{p}^{2}$ = 0.077; *β* = -0.45; *p* = 0.03) predicted symptom improvements. Current psychosis at baseline predicted high symptom levels at follow-up ($\eta_{p}^{2}$ = 0.17; *β* = 0.81; *p* = 0.001).

**Table 5:** Analysis of variance with parameter estimates showing baseline variables that were associated with change of the Global Severity Index on the revised version of the Symptom Checklist with 90 items at follow-up.

		ANOVA					Parameter estimates		
	Reference category	Sum of squares	*F*	df	*p*-value	Partial eta-squared	Coefficient B*	Standard error	T
**Socio-demographic variables**									
Sex	male	1.75	3.43	1	n.s.	0.055	-0.39	0.21	-1.85
Educational level^a^	high	0.17	0.34	1	n.s.	0.006	-0.12	0.21	-0.58
**Variables related to imprisonment**								
Working^b^	yes	5.42	10.61	1	0.002	0.152	-0.71	0.22	-3.26
Intervals out of prison during follow-up period^c^	yes	2.52	4.94	1	0.030	0.077	-0.45	0.20	-2.22
Legal status^d^	sentenced	0.87	0.17	1	n.s.	0.007	-0.10	0.26	-0.41
Mental health treatment^e^	yes	0.06	0.11	1	n.s.	0.002	0.75	0.22	0.34
**Psychiatric disorders at baseline**									
Depression^f^	yes	4.79	9.39	1	0.003	0.137	-0.67	0.22	-3.06
Anxiety disorders^g^	yes	0.30	0.58	1	n.s.	0.010	-0.17	0.23	-0.76
Psychotic disorders^h^	yes	6.18	12.11	1	0.001	0.170	0.81	0.23	3.48
PD	yes	2.01	3.94	1	n.s.	0.063	-0.48	0.24	-1.98
IDUD	yes	1.58	3.09	1	n.s.	0.050	0.37	0.21	1.76
AUD	yes	0.01	0.02	1	n.s.	0	-0.03	0.21	-0.14

^a^Following the International Standard Classification of Education, dichotomized into low (0–1) and high (2–6)

^b^working for remuneration or studying

^c^at least one interval outside prison during the three year follow-up

^d^sentenced or on remand

^e^including treatment by general physician, psychiatrist or psychologist for mental health problems or any use of prescribed psychotropic medication

^f^including major depression, recurrent depression

^g^including current panic disorder, agoraphobia, social anxiety disorder, generalized anxiety disorder, obsessive-compulsive disorder, posttraumatic stress disorder

^h^including current psychotic disorder, current psychotic mood disorder; PD, personality disorder (including borderline personality disorder and antisocial personality disorder); IDUD = illicit drug use disorder (including drug dependence and drug abuse); AUD = alcohol use disorder (including alcohol dependence and alcohol abuse)

n.s. = non-significant.

*Coefficient B of the parameter estimates >0 indicates higher mean GSI for the reference category (less symptom improvement than expected), Coefficient B <0 indicates lower mean GSI for the reference category (more symptom improvement than expected).

## Discussion

### Main findings

The prevalence of major mental illnesses including depression and psychotic disorders was significantly lower at the 3-year follow-up than at intake into prison. Psychological symptom levels improved between baseline and follow-up in all domains. Working for remuneration or studying during imprisonment showed the strongest association with symptom improvement at follow-up. People with depression at baseline were likely to show marked improvements of psychological symptoms, whereas people with psychosis at baseline had the smallest symptom improvements at follow-up.

### Strengths and limitations

To our knowledge, this is the first study of its kind in South America and has several methodological strengths. It follows the course of psychiatric disorders in a random admission sample. The sample is representative of people consecutively admitted to the penal justice system who are continuously or intermittently imprisoned after three years. The response rate at follow-up was very high (94%). The study used standardized and validated diagnostic instruments applied by trained researchers.

The major limitation of this study is the relatively small sample size, which limits the power of the statistical tests. Also, comorbidity was not taken into account, and diagnostic groups may be overlapping, potentially complicating the prediction of long-term courses. The sample was not representative for the total prison population. Corresponding to the longitudinal long-term follow-up design of the study, we only recruited people continuously or intermittently involved with the penal justice system over a relatively long time period. The study design over-represents prisoners with long sentences and high turnover rates compared to the total prison population.

### Interpretations and comparison with the literature

The prevalence of major depression decreased mostly without specific therapeutic interventions. These findings are in line with other studies from prison samples in high-income countries. Longitudinal studies in such samples indicated that levels of depression decrease during imprisonment [6, 7]. This seems to hold true in this South American context with a more poorly resourced prison system. Studies in prison populations with follow-up periods of one year or more after intake reported that more than half of the individuals with major depression show remission at follow-up [11, 12]. This is similar to the results in a naturalistic study on the course of major depression in the community that found 40% still had depression after one year [22]. Even though we found lower prevalence of major depression at follow-up in this study, there are substantial numbers of people who still experience depression after three years, and other new cases of depression that need to be considered for service development [23].

The prevalence of psychosis was lower at follow-up, but symptom levels remained high in this group. People with psychosis at intake should be prioritized with respect to screening and treatment development since the course of their disorders may be relatively unfavorable in the prison environment.

Substance use disorders constitute an important health problem among prisoners [24–26]. They increase the risk of reoffending on release [27] and mortality [28, 29]. Prevalence rates of substance use disorders have been shown to be especially high at admission to the penal justice system [30]. Interestingly, individuals with dependence disorders were described to have lower risk of psychiatric disorders in prison, and may even benefit from the prison environment [31]. Our data did not show significantly lower rates of substance use disorders at follow-up. There were 10 new cases of illicit drug use disorders (IDUD) and 7 new cases of alcohol use disorders (AUD) among people who had not shown those disorders at intake to prison. On average, individuals with AUD showed the largest improvement with regard to both symptom levels and suicide risk. This appears to be a consequence of the alcohol ban inside prison, which is probably more effective than the ban on illicit drugs. We did not see the same level of symptom improvement for people with IDUD, which suggests that illicit drug use is easier to maintain during imprisonment. The controlled prison environment may therefore provide an opportunity for successful therapeutic interventions for substance use disorders.

Anxiety disorders remain high during imprisonment, as well as personality disorders which we expected to be relatively stable.

Risk for suicide in prison is high [32] and has been linked to clinical, environmental and socio-demographic factors [33–35], and more specific factors such as separation from family due to imprisonment [36]. At follow-up mean risk of suicide decreased which is in line with reduced symptom scores and the decrease of major depression and psychotic disorders. Despite a decrease in the number of prisoners who reported a moderate to high suicide risk, there was an increase in people reporting a low suicide risk at follow-up compared to baseline. Furthermore, 15% of the sample attempted suicide during the 3-year follow-up period, of which the majority had a high suicide risk at admission. This indicates that assessment of mental health and risk factors for suicide at admission is vital, and subsequent interventions for suicide prevention are needed in newly imprisoned populations [37].

Global distress and all nine dimensions of psychopathology covered by the SCL-90-R improved significantly during the three years of follow-up, but rates remained above those of the general public (as established in a study in Chile [38]). Longitudinal studies with shorter follow-up periods have applied the SCL-90 to imprisoned people [10, 21] and showed higher symptom scores at imprisonment than after five days and four weeks respectively. Since the symptom scores on the SCL-90 reflect mental states in the week prior to the assessment, studies conducted on the day of intake may rather reflect mental health states prior to imprisonment. The scores after five days, reflect health states during the first week of imprisonment suggesting symptom improvements at very early stages of imprisonment. This overall trend seems to continue for most prisoners over longer terms of imprisonment. A significant decrease of symptom scores in individuals with depression was shown by a British study that compared the decrease of psychological symptom levels between imprisoned individuals with and without mental illness at confinement [7].

Short-term prisoners had lower levels of distress than long-term prisoners, which has also been observed in another sample of imprisoned individuals [39]. The international literature points to symptom improvement in sentenced individuals as compared to those who remain on remand [7]. Whereas all participants in our study were on remand at baseline, the majority was sentenced at follow-up. The sentencing may improve the sense of control and predictability and thus have positive impact on mental health. Meaningful occupation seems to be an important factor for mental health outcomes, and is part of the UN Minimum Rules for treatment of imprisoned individuals to offer remunerated work, vocational training or education aimed at the full development of the human personality [40]. Even though imprisoned individuals would deliberately choose work or education over doing nothing [41], in this study only one out of four had worked or attended educational programs during the three year follow-up. Beyond the mental health benefits during imprisonment, work and educational programs have positive effects on rehabilitation and recidivism after release [42, 43]. Given the positive interaction of work and mental health symptoms, further expansion of current programs is warranted.

## References

[1] World Prison Population List (eleventh edition) [Internet]. Institute for Criminal Policy Research. 2016 [cited 26.03.2016]. Available from: http://www.prisonstudies.org/sites/default/files/resources/downloads/world_prison_population_list_11th_edition.pdf.

[2] Mundt AP, Chow WS, Arduino M, Barrionuevo H, Fritsch R, Girala N, et al Psychiatric Hospital Beds and Prison Populations in South America Since 1990. JAMA Psychiatry. 2015;72(2):112–7. 10.1001/jamapsychiatry.2014.2433 25471050

[3] Chile | World Prison Brief [Internet]. Institute for Criminal Policy Research. 2017 [cited 2 March 2018]. Available from: http://prisonstudies.org/country/chile.

[4] Fazel S, Seewald K. Severe mental illness in 33 588 prisoners worldwide: systematic review and meta-regression analysis. Br J Psychiatry. 2012;200(5):364–73. Epub 2012/05/03. 10.1192/bjp.bp.111.096370 . 22550330

[5] Mundt AP, Kastner S, Larraín S, Fritsch R, Priebe S. Prevalence of mental disorders at admission to the penal justice system in emerging countries: a study from Chile. Epidemiol Psychiatr Sci. 2015;25(05):441–9. 26088528 10.1017/S2045796015000554 PMC7137584

[6] Walker J, Illingworth C, Canning A, Garner E, Woolley J, Taylor P, et al Changes in mental state associated with prison environments: a systematic review. Acta Psychiatr Scand. 2014;129(6):427–36. 10.1111/acps.12221 . 24237622

[7] Hassan L, Birmingham L, Harty MA, Jarrett M, Jones P, King C, et al Prospective cohort study of mental health during imprisonment. Br J Psychiatry. 2011;198(1):37–42. 10.1192/bjp.bp.110.080333 . 21200075

[8] Hurley W, Dunne MP. Psychological Distress and Psychiatric Morbidity in Women Prisoners. Aust N Z J Psychiatry. 1991;25(4):461–70. 10.3109/00048679109064439 1793416

[9] Harding T, Zimmermann E. Psychiatric symptoms, cognitive stress and vulnerability factors. A study in a remand prison. The British Journal of Psychiatry. 1989;155(1):36–43. 10.1192/bjp.155.1.36 2605430

[10] Taylor PJ, Walker J, Dunn E, Kissell A, Williams A, Amos T. Improving mental state in early imprisonment. Crim Behav Ment Health. 2010;20(3):215–31. 10.1002/cbm.774 . 20549784

[11] Zamble E, Porporino F. Coping, Imprisonment, and Rehabilitation: Some Data and their Implications. Criminal Justice and Behavior. 1990;17(1):53–70. 10.1177/0093854890017001005

[12] Baier A, Fritsch R, Ignatyev Y, Priebe S, Mundt AP. The course of major depression during imprisonment—A one year cohort study. J Affect Disord. 2016;189:207–13. 10.1016/j.jad.2015.09.003 . 26451505

[13] UNESCO Institute for Statistics. International Standard Classification of Education ISCED Montreal2011 [28 May 2018]. Available from: http://ec.europa.eu/eurostat/statistics-explained/index.php/International_Standard_Classification_of_Education_%28ISCED%29.

[14] Black DW, Arndt S, Hale N, Rogerson R. Use of the Mini International Neuropsychiatric Interview (MINI) as a screening tool in prisons: results of a preliminary study. J Am Acad Psychiatry Law. 2004;32(2):158–62. Epub 2004/07/30. . 15281417

[15] Fotiadou M, Livaditis M, Manou I, Kaniotou E, Xenitidis K. Prevalence of mental disorders and deliberate self-harm in Greek male prisoners. Int J Law Psychiatry. 2006;29(1):68–73. 10.1016/j.ijlp.2004.06.009 16266748

[16] Gunter TD, Arndt S, Wenman G, Allen J, Loveless P, Sieleni B, et al Frequency of mental and addictive disorders among 320 men and women entering the Iowa prison system: use of the MINI-Plus. J Am Acad Psychiatry Law. 2008;36(1):27–34. Epub 2008/03/21. . 18354120

[17] Maccio A, Meloni FR, Sisti D, Rocchi MB, Petretto DR, Masala C, et al Mental disorders in Italian prisoners: results of the REDiMe study. Psychiatry Res. 2015;225(3):522–30. 10.1016/j.psychres.2014.11.053 . 25534756

[18] Pondé MP, Freire AC, Mendonça MS. The prevalence of mental disorders in prisoners in the city of Salvador, Bahia, Brazil. J Forensic Sci. 2011;56(3):679–82. Epub 2011/02/11. 10.1111/j.1556-4029.2010.01691.x . 21306379

[19] First MB, Gibbon M, Spitzer RL, Williams JBW, Benjamin LS. Structured Clinical Interview for DSM-IV Axis II Personality Disorders, (SCID-II). American Psychiatric Press 1997.

[20] Ignatyev Y, Fritsch R, Priebe S, Mundt AP. Psychometric properties of the symptom check-list-90-R in prison inmates. Psychiatry Res. 2016;239:226–31. 10.1016/j.psychres.2016.03.007 . 27031592

[21] Gibbs JJ. Symptoms of Psychopathology among Jail Prisoners: The Effects of Exposure to the Jail Environment. Criminal Justice and Behavior. 1987;14(3):288–310. 10.1177/0093854887014003003

[22] Chin WY, Chan KT, Lam CL, Wan EY, Lam TP. 12-Month naturalistic outcomes of depressive disorders in Hong Kong's primary care. Fam Pract. 2015;32(3):288–96. 10.1093/fampra/cmv009 25746447 PMC4445136

[23] Bukh JD, Bock C, Vinberg M, Kessing LV. The effect of prolonged duration of untreated depression on antidepressant treatment outcome. J Affect Disord. 2013;145(1):42–8. 10.1016/j.jad.2012.07.008 . 22854096

[24] Fazel S, Bains P, Doll H. Substance abuse and dependence in prisoners: a systematic review. Addiction. 2006;101(2):181–91. 10.1111/j.1360-0443.2006.01316.x . 16445547

[25] Fazel S, Yoon IA, Hayes AJ. Substance use disorders in prisoners: an updated systematic review and meta-regression analysis in recently incarcerated men and women. Addiction. 2017;112(10):1725–39. 10.1111/add.13877 28543749 PMC5589068

[26] Mundt AP, Baranyi G, Gabrysch C, Fazel S. Substance Use During Imprisonment in Low- and Middle-Income Countries. Epidemiol Rev. 2018 10.1093/epirev/mxx016 . 29584860 PMC5982797

[27] Chang Z, Larsson H, Lichtenstein P, Fazel S. Psychiatric disorders and violent reoffending: a national cohort study of convicted prisoners in Sweden. Lancet Psychiatry. 2015;2(10):891–900. 10.1016/S2215-0366(15)00234-5 26342957 PMC4629414

[28] Chang Z, Lichtenstein P, Larsson H, Fazel S. Substance use disorders, psychiatric disorders, and mortality after release from prison: a nationwide longitudinal cohort study. Lancet Psychiatry. 2015;2(5):422–30. 10.1016/S2215-0366(15)00088-7 26360286 PMC4579558

[29] Zlodre J, Fazel S. All-cause and external mortality in released prisoners: systematic review and meta-analysis. Am J Public Health. 2012;102(12):e67–75. 10.2105/AJPH.2012.300764 23078476 PMC3519300

[30] Mir J, Kastner S, Priebe S, Konrad N, Ströhle A, Mundt AP. Treating substance abuse is not enough: Comorbidities in consecutively admitted female prisoners. Addict Behav. 2015;46(C):25–30. 10.1016/j.addbeh.2015.02.016 . 25770695

[31] Andersen HS, Sestoft D, Lillebæk T, Gabrielsen G, Hemmingsen R. A longitudinal study of prisoners on remand. Repeated measures of psychopathology in the initial phase of solitary versus nonsolitary confinement. Int J Law Psychiatry. 2003;26(2):165–77. 12581753 10.1016/s0160-2527(03)00015-3

[32] Fazel S, Grann M, Kling B, Hawton K. Prison suicide in 12 countries: an ecological study of 861 suicides during 2003–2007. Soc Psychiatry Psychiatr Epidemiol. 2011;46(3):191–5. Epub 2010/02/09. 10.1007/s00127-010-0184-4 . 20140663

[33] Fazel S, Cartwright J, Norman-Nott A, Hawton K. Suicide in prisoners: a systematic review of risk factors. J Clin Psychiatry. 2008;69(11):1721–31. Epub 2008/11/26. . 19026254

[34] Baillargeon J, Penn JV, Thomas CR, Temple JR, Baillargeon G, Murray OJ. Psychiatric Disorders and Suicide in the Nation's Largest State Prison System. J Am Acad Psychiatry Law. 2009;37:188–93. 19535556

[35] Marzano L, Hawton K, Rivlin A, Fazel S. Psychosocial influences on prisoner suicide: a case-control study of near-lethal self-harm in women prisoners. Soc Sci Med. 2011;72(6):874–83. Epub 2011/02/25. 10.1016/j.socscimed.2010.12.028 . 21345561

[36] Krüger S, Priebe S, Fritsch R, Mundt AP. Burden of separation and suicide risk of prisoners with minor children. Int J Law Psychiatry. 2017;52:55–61. 10.1016/j.ijlp.2017.03.004 . 28395894

[37] Pratt D, Tarrier N, Dunn G, Awenat Y, Shaw J, Ulph F, et al Cognitive-behavioural suicide prevention for male prisoners: a pilot randomized controlled trial. Psychol Med. 2015:1–11. 10.1017/S0033291715001348 . 26165919 PMC4682193

[38] Gempp Fuentealba R, Avendaño Bravo C. Datos normativos y propiedades psicométricas del SCL-90-R en estudiantes universitarios chilenos. Terapia Psicológica. 2008;26:39–58.

[39] Otte S, Vasic N, Nigel S, Streb J, Ross T, Spitzer C, et al Different yet similar? Prisoners versus psychiatric patients—A comparison of their mental health. Eur Psychiatry. 2017;44:97–103. 10.1016/j.eurpsy.2017.04.006 . 28628826

[40] Standard Minimum Rules for the Treatment of Prisoners. In: United Nations Justice Section DfO, editor. Nelson Mandela Rules. General Assembly resolution 70/175, annex, ed. Vienna International Centre, P.O. Box 500, 1400 Vienna, Austria: UN Office on Drugs and Crime; 2015.

[41] Batchelder JS, Pippert JM. Hard time or idle time: factors affecting inmate choices between participation in prison work and education programs. Prison J. 2002;82(2):269–80. 10.1177/003288550208200206

[42] Saylor WG, Gaes GG. The Post-Release Employment Project. Federal Prisons Journal 1992;2(4):33–6.

[43] Vacca JS. Educated Prisoners Are Less Likely to Return to Prison The Journal of Correctional Education. 2004;55(4):297–305.

