# Correction: Mental disorders and mental health symptoms during imprisonment: A three-year follow-up study

**DOI:** 10.1371/journal.pone.0231593

**Published:** 2020-04-03

**Authors:** Caroline Gabrysch, Rosemarie Fritsch, Stefan Priebe, Adrian P. Mundt

In the Results section of the Abstract, the third sentence is incorrect. The correct sentence is: The mean GSI improved from 1.6 (SD 0.84) at intake to 1.2 (SD 0.82) at follow-up (p<0.001).

In the Mental disorders at admission and at follow-up section of the Results, the second sentence is incorrect. The correct sentence is: Out of 47 (64%) individuals with major depression at baseline, 19 (26%) still met the diagnostic criteria at follow-up, and 28 (38%) had been remitted at follow-up and there were four new cases.

In the Mental disorders at admission and at follow-up section of the Results, the fourth sentence is incorrect. The correct sentence is: The prevalence of current psychotic disorders decreased from 22 (30%) at baseline to 10 (14%) at follow-up (p = 0.008).

There are errors in Table 2. Please see the correct Table 2 here.

**Table 2:** The prevalence of psychiatric disorders (diagnostic groups) among prisoners at baseline and at three-year follow-up.

	Baseline			Follow-up			McNemar test			
	N = 73	Prevalence in % (CI)		N = 73	Prevalence in % (CI)		Cases at baseline and follow-up (*n)*	New cases at follow-up (*n)*	Remitted cases at follow-up (*n)*	p-Value
Depression^a^	47	64	(53.4–75.3)	23	32	(20.5–42.5)	19	4	28	<0.001
Anxiety disorders^b^	32	44	(31.5–56.2)	27	37	(26–47.9)	18	9	14	n.s.
Psychotic disorders^c^	22	30	(19.2–39.7)	10	14	(6.8–23.3)	7	3	15	0.008
PD	52	71	(60.3–82.2)	51	70	(58.9–80.8)	42	9	10	n.s.
IDUD	47	64	(53.4–75.3)	36	50	(38.4–61.6)	26	10	21	n.s.
AUD	22	30	(19.2–41.1)	13	18	(9.6–27.4)	6	7	16	n.s.

^a^Including major depression, recurrent major depression

^b^including current panic disorder, agoraphobia, social anxiety disorder, generalized anxiety disorder, obsessive-compulsive disorder, posttraumatic stress disorder

^c^including current psychotic disorder, current psychotic mood disorder

PD = personality disorder (including borderline personality disorder and antisocial personality disorder)

IDUD = illicit drug use disorder (including drug dependence and drug abuse)

AUD = alcohol use disorder (including alcohol dependence and alcohol abuse)

n.s. = non-significant.
